# MicroRNA-217 Promotes Angiogenesis of Human Cytomegalovirus-Infected Endothelial Cells through Downregulation of SIRT1 and FOXO3A

**DOI:** 10.1371/journal.pone.0083620

**Published:** 2013-12-20

**Authors:** Shanchao Zhang, Lei Liu, Ruijin Wang, Houzhen Tuo, Yanjun Guo, Li Yi, Dexin Wang, Jiawei Wang

**Affiliations:** Department of Neurology, Beijing Friendship Hospital, Capital Medical University, Xicheng District, Beijing, China; University of Regensburg, Germany

## Abstract

Human cytomegalovirus(HCMV) infection has been shown to contribute to vascular disease through the induction of angiogenesis. However, the role of microRNA in angiogenesis induced by HCMV infection remains unclear. The present study was thus designed to explore the potential effect of miR-1217 on angiogenesis and to disclose the underlying mechanism in endothelial cells. We found that HCMV infection of endothelial cells(ECs) enhanced expression of miR-217 and reduced SIRT1 and FOXO3A protein level in 24 hours post infection(hpi). Transfection of miR-217 inhibitor not only depressed cellular migration and tube formation induced by HCMV infection, but also enhanced SIRT1 and FOXO3A protein expression. Additionally, luciferase assay confirmed that miR-217 directly targeted FOXO3A mRNA 3`UTR. Furthermore, pretreatment with resveratrol depressed motility and tube formation of HCMV-infected ECs, which could be reversed by SIRT1 siRNA. Similarly, delivery of FOXO3A overexpression lentivirus suppressed proliferative rate, migration and tube formation of HCMV-infected ECs, which reversed by transfection of FOXO3A siRNA. In summary, HCMV infection of endothelial cells induces angiogenesis by both of miR-217/SIRT1 and miR-217/FOXO3A axis.

## Introduction

Epidemiological and animal studies have associated human cytomegalovirus(HCMV) infection with the acceleration of vascular disorders such as coronary artery disease, transplant vascular sclerosis, arterial stenosis and atherosclerosis [1], [2]. The ability of the virus to infect vascular endothelial cells(ECs) and to dysregulate their gene expression profiles, their physiological states of activation and differentiation and their interactions with other cell types is thought to be crucial in HCMV pathogenesis [3], [4]. There now are mounting evidences that HCMV infection is correlative with the development of chronic diseases that involve angiogenesis. HCMV-infected cells directly induce angiogenesis by secreting VEGF and/or other angiogenic factors [5]. Factors secreted from infected cells(secretome) have direct effects of promoting tube formation and stabilization [6], [7]. HCMV infection of endothelial cells induces an angiogenic response through viral binding to the EGF receptor and β_1_ and β_3_ integrins [8].

MicroRNAs (miRNA) are small, non-coding RNAs of approximately 22 nucleotides encoded within the genome and derived from endogenous short hairpin precursors. The mature miRNAs negatively regulate gene expression by targeting specific messenger RNAs(mRNAs) for cleavage or translational repression [9]. A growing body of evidence suggests that they are involved in the control of a wide range of physiological pathways, such as development, differentiation, growth, and metabolism, as well as in disease conditions [10], [11]. Recent evidence has implicated overall miRNA levels in regulating angiogenesis and endothelial function [12], [13]. In the present study, we found that miR-217 is progressively expressed in HCMV-infected ECs. More importantly, we demonstrated that HCMV-mediated induction of miR-217 results in SIRT1 and FOXO3A suppression and subsequently promotes angiogenesis of endothelial cells.

## Materials and Methods

### Cells and Virus Preparation

Human umbilical vein endothelial cell(HUVEC) was purchased from ScienCell. The cells were cultured in endothelial cell medium supplemented with 10%FBS, 1% endothelial cell growth supplement(ECGS), 100IU/ml penicillin, 0.1mg/ml streptomycin(ScienCell), and used at passages 2 to 6. A human embryonic fibroblast cell line(MRC-5) was obtained from Xie He Medical University. Cells was cultured in MEM(HyClone) supplemented with 10%FBS, 10mM L-glutamine, 1mM sodium pyruvate, 1%NEAA(Non-essential Amino Acid)(100X), 100IU/ml penicillin and 0.1mg/ml streptomycin. Viral titers were determined by cytopathic effect on MRS-5 cells. The human kidney cell line 293T, obtained from Xie He Medical University, was cultured in high glucose DMEM supplemented with 10% FBS, 100IU/ml penicillin and 0.1mg/ml streptomycin. All cells were incubated in a humidified atmosphere of 5% CO2 at 37°C. HCMV laboratory strain Towne, purchased from ATCC, was produced by infected MRC-5 cells. Infected cell supernatants were recovered at maximum cytopathic effect and stored at −80°C until later use. Virus titers were determined by TCID50 assay. Mock-infected inocula was prepared in an identical fashion, except that cell monolayers were not infected with HCMV. HUVECs were infected at MOI of 5 based on infectivity of MRC-5. Immediate-early protein 72 was used to monitor HCMV infection(Fig. S1).

### Quantitative Reverse Transcription-Polymerase Chain Reaction(qRT-PCR) Analysis

Total RNA from the cells was extracted using miRNeasy Micro Kit according to the manufacture protocol(Qiagen). RNA was reversely transcribed using miScript ⨿ RT Kit(Qiagen). The amplification were carried out in triplicate by miScript SYBR® Green PCR Kit(Qiagen) on a 7900HT Real-Time PCR system(Applied Biosystems) according to the manufacturer's instructions. The expression of miR-217 was normalized to U6. SIRT1 and FOXO3A mRNA were normalized to β-actin. Primer sequences for specific genes are presented as followed. The primers of miR-217 and U6 were from miScript Primer Assays(Qiagen).

SIRT1 Forward: GTATTTATGCTCGCCTTGCTG

SIRT1 Reverse: TGACAGAGAGATGGCTGGAA

FOXO3A Forward: GCAGACCATCCAAGAGAACAA

FOXO3A Reverse: TGTGGCTAAGTGAGTCCGAAG

ß-actin Forward: AGCACAATGAAGATCAAGATCAT

ß-actin Reverse: ACTCGTCATACTCCTGCTTGC

The primers were purchased from Songon (Shanghai, China).

### Western Blot Analysis

Cells were lysed in RIPA buffer containing protease inhibitor mixture(Roche). After incubation at 4°C for 30 minutes, soluble proteins were collected by centrifugation at 12000rpm for 15 minutes. Supernatants were analyzed for protein concentration with a Pierce protein assay kit and stored at −80°C. Proteins were separated on 10% SDS-polyacrylamide gel, and then transferred to Immobilon-P membranes(Millipore). The filters were immunostained with rabbit monoclonal against SIRT1(1∶1000, Cell Signaling Technology), and FOXO3A(1∶1000, Cell Signaling Technology), and antibody against β-actin(1∶1000, Cell Signaling Technology) as an internal control. The immunocomplexes were detected with secondary antibody conjugated to horseradish peroxidase(1∶10,000, Santa Cruz Biotechnology) and visualized with the use of a Immobilon™ Western Chemiluminescent Kit(Millpore).

### siRNA and microRNA transfection

HUVECs were seeded in six-well plates to ensure that they would reach 60%–70% confluence the following day. For small interfering RNA (siRNA)-mediated gene knockdown, 100 nmol/L of SIRT1 or FOX3A siRNA(Santa Cruz) or 100 nmol/L scramble siRNA(Santa Cruz) was transfected into cells. For knockdown of miR-217, cells were transfected with 100 nmol/L of miR-217 inhibitor(Ambion). All siRNAs and miRNAs were transfected into endothelial cells with Lipofectamine™ RNAiMax according to the manufacturer's protocol. After 24 hours of transfection for SIRT1 siRNA or 48 hours for FOXO3A siRNA and miR-217, cells were infected with HCMV or mock for 24 hours. After 24 hours of incubation, the cells were processed for further analysis.

### Lentivirus Vector Production and Infection

To construct the lentivirus vector Lv-FOXO3A which overexpresses FOXO3A, a fragment encoding the FOXO3A sequence plus 2065 bp at both 5`- and 3`-flanking regions was amplified with the primers 5`-GAGGATCCCCGGGTACCGGTCGCCACCATGGCAGAGGCACCGGCTTC-3`(forward) and 5`-TCACCATGGTGGCGACCGGGCCTGGCACCCAGCTCTGÀ (reverse) by PCR from human genomic DNA and then cloned into the AgeI sites of Ubi-MCS-EGFP vector(Genechem, Shanghai, China). For lentiviral infection, cells were plated at a concentration of 1×10^5^ cells in six well plate after overnight culture and were then infected at MOI of 20 in the presence of polybrene (5 ug/ml) for 10 hours. Infected cells were continued to culture for 72 hours with 10% FBS medium. The protein level of FOXO3A was detected by western blot.

### Cell Proliferation Assay

CCK-8 assays(Dojindo) were performed by using the manufacture's protocol. Briefly, cells were seeded on 96-well plates at 2×10^3^ cells/well before mock or HCMV infection. The CCK-8 assay was performed after 72hpi. After 2 hours of incubation with culture medium containing the CCK-8 reagent, the absorbance was read at 450 nm using a microplate enzyme-linked immunosorbent assay reader(Bio-Rad).

### In vitro Cell Migration Assay

To determine the migration of endothelial cells, transwell migration experiments were performed in a 24-well modified Boyden chamber(8μm, Corning). Approximately 5×10^4^ cells in 0.2 ml serum-free medium were added into each filter of the upper chamber. The lower chamber was filled with 0.6 ml complete culture medium. Following 24 hours of incubation, the nonmigrating cells on the upper side of the chamber were mechanically removed. The cells on the lower side of the chamber were fixed in 4% paraformaldehyde for 15 minutes. For quantification, cell nuclei were stained with DAPI and microscopically observed and counted in five representative fields.

### Tube Formation Assay

HUVECs(1.2×10^4^/well) were cultured in a 96-well plate coated with 60 µL Matrigel(BD Biosciences). Tube formation was defined as a tube-like structure exhibiting a length four times its width [13]. Tube morphology was quantified after 24 hours in 5 random microscopic fields with a computer-assisted microscope(Nikon).

### Vector Construction and Reporter Assays

Prediction of miR-217 binding sites was performed using TargetScan software(http://www.targetscan.org/). Sequences including 3`UTR of SIRT1 containing the SIRT1-miR-217 response elements contained 5`-AUUUAUUUGGCUACACUAAAGA***AUGCAGUA***-3`(the bold and italic nucleotides are miR-217 binding sequences) [16]. To construct FOXO3A 3` UTR plasmid, a wild-type 3`-UTR fragment of human FOXO3A mRNA (Genbank accession NM_001455), whose sequences contained 5`-AGTGGGTAACATTTT***ATGCAGT***T-3` and 5`-CCAAATGAAATAGAG***ATGCAGTA***-3`(these bold and italic nucleotides are two putative miR-217 binding sites), was synthesized and cloned into the XbaI site just downstream of the firefly luciferase structural gene in SV40-Luc_firefly-MCS vector(Genechem, Shanghai, China). The corresponding mutant construct was created by mutating the seed regions of the miR-217-binding sites. All plasmids(wild-type and mutant) were verified by sequencing. After sequence verification, we obtained plasmid clones containing correctly oriented inserts. Five thousand cells per well were seeded onto 96-well plates 24 hours prior to transfection. Cells were transfected with miR-217 mimics(50nmol) or negative control(50 nmol)(Ambion). 24 hours after transfection, the cells were co-transfected with constructed wild type or mutated vector(firefly luciferase) and internal control pRL-TK vector(Renilla luciferase, promega) using Lipofectamine 2000(Invitrogen). 48 hours after plasmid vector transfection, cell lysates were prepared with Dual-Glo® Luciferase Reagent(Promega), and luciferase activities were measured by using a Dual-Glo™ Luciferase Assay System(Promega). The luminescence intensity of firefly luciferase was normalized to that of Renilla luciferase.

### Statistical analysis

Data were expressed as means ± SDs. Two treatment groups were compared by the unpaired Students t test; one-way ANOVA was performed for serial analysis. A value of P<0.05 was considered statistically significant.

## Results

### 1. 1. HCMV infection downregulates endogenous SIRT1 and FOXO3A in HUVECs

**Figure 1:**
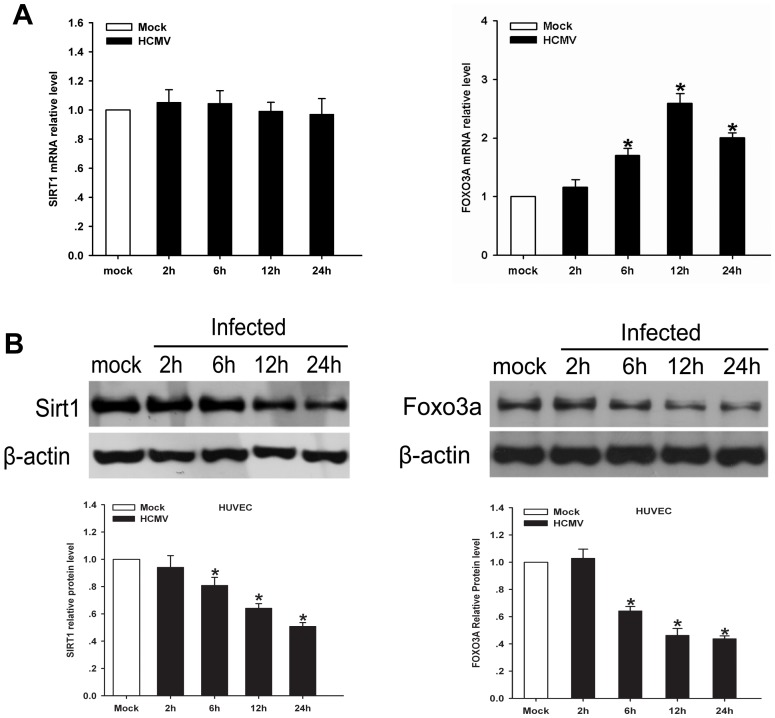
HCMV infection of endothelial cells reduces SIRT1 and FOX3A expression in endothelial cells. A. QRT-PCR analysis of SIRT1 and FOXO3A mRNA in HUVECs at the indicated times after infection and the relative amount of mRNA was normalized to β-actin. SIRT1 or FOXO3A mRNA level from mock infected ECs serves as reference. B. Western blot analysis of SIRT1 and FOXO3A protein level in HUVECs was performed using equal protein loading of mock-infected and HCMV-infected. The histogram shows relative density of SIRT1 and FOX3A compared to mock infection. Values are expressed as mean±SD from three independent experiments, *p <0.05 vs. controls.

SIRT1, a nicotinamide adenine dinucleotide(NAD^+^)-dependent histone deacetylase, has been reported to highly express in the vasculature during blood vessel growth and control the angiogenic activity of endothelial cells [14]. FOXO3A, one of forkhead transcription factors, has been demonstrated to involve in angiogenesis and postnatal neovascularization [15]. Recent study demonstrates that HCMV infection of ECs directly induces cellular proliferation, migration and new capillary tube formation [8]. To test whether SIRT1 or FOXO3A is involved in angiogenesis induced by HCMV infeciton, qRT-PCR analyses to examine mRNA level of SIRT1 and FOXO3A were performed on mock or HCMV-infected HUVECs. During 24hpi, mRNA level of SIRT1 remained unaffected(Fig. 1A), and FOXO3A mRNA expression continued to accumulate in a time-dependent manner, with peak level being detected at 12hpi(Fig. 1A). We next examined the protein level of SIRT1 or FOXO3A of infected-HUVECs. As shown in Fig. 1B, our experimental data presented that the expression of SIRT1 and FOXO3A protein was depressed during 24hpi.

### 2. 2. SIRT1 and FOXO3A mediate angiogenesis of HUVECs exposed to HCMV infection

**Figure 2:**
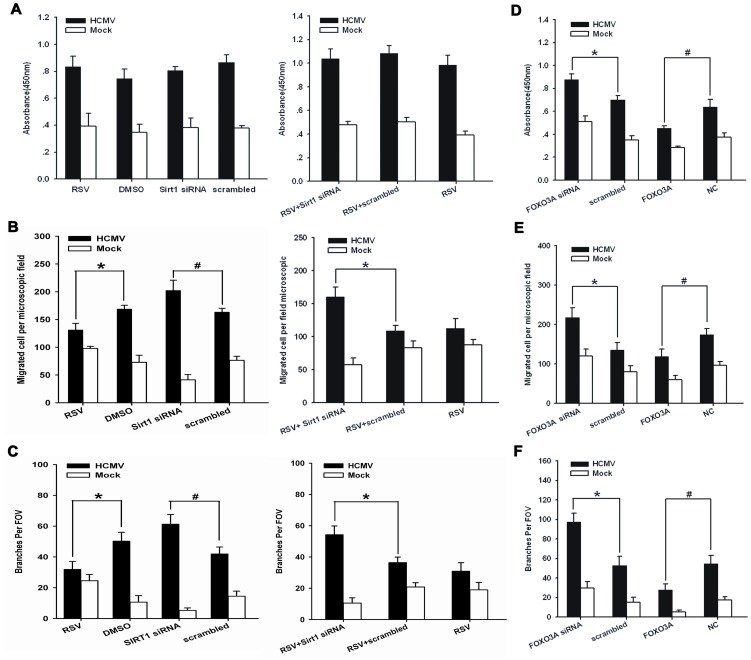
Effect of SIRT1 and FOXO3A on the angiogenic activity of endothelial cells. A. (1) ECs were treated for four different treatments before infection: 1. resveratrol(20umol) for 4 hours; 2. DMSO;3. SIRT siRNA(100nmol) for 24 hours; 4. negative control. (2) Cells were transfected with SIRT1 siRNA or control siRNA(100 nmol) and then treated with resveratrol(20umol) before infection. Proliferation was measured with CCK-8 assay at 72hpi. B. Cell migration analysis of ECs that were treated with the same methods as those used in Fig. 2A. C. Cell tube formation analysis of endothelial cells that were treated with the same methods as those used in Fig. 2A. D. Cell proliferation rate analysis in infected ECs in the presence of FOXO3A siRNA or FOXO3A overexpressing lentivirus vector at 72hpi. E. Cell migration at 24 hours using the same methods as those used in Fig. 2D. F. Matrigel tube formation analysis using the same methods as those used in Fig. 2D. Data represents the mean value of three independent experiments, presented as Means±SD. *P and **^#^**P<0.05. hpi, hour post infection; RSV, resveratrol. FOXO3A, foxo3a lentivirus vector; NC, normal control vector.

We next evaluated whether HCMV infection mediates angiogenic response of ECs through SIRT1. To test this hypothesis, cells were pretreated with either SIRT1 activator resveratrol or SIRT1 siRNA before HCMV infection. As seen in Fig. S2A, SIRT1 expression was reduced by siRNA. Cell proliferation in either HCMV or mock infection remained unchanged with treatment of resveratrol or SIRT1 siRNA(Fig. 2A). However, in HCMV-infected ECs, cell motility and tube formation were significantly(P<0.05) abrogated when treated with resveratrol, but enhanced when transfected with SIRT1 siRNA(Fig. 2B and 2C and Fig. S2B and S2C). By contrast, motility and tube formation ability of mock-infected cells was enhanced by resveratrol, and depressed by SIRT1 siRNA.

We next tested whether FOXO3A modulates the angiogenic response induced by HCMV infection. Downregulation of FOXO3A protein using siRNA can be seen in Fig. S2D. Fig. S2E indicated that lentiviral overexpression of FOXO3A protein significantly induced protein expression at multiplicity of infection 20. Our experimental data demonstrated that overexpression of FOXO3A significantly impaired proliferation, migration and tube formation of either HCMV or mock-infected ECs whereas transfection of FOXO3A siRNA enhanced these angiogenic responses(Fig. 2D, 2E, 2F and Fig. S2F and S2G).

### 3. 3. Downregulation of miR-217 attenuates angiogenic response by upregulation of SIRT1 and FOXO3A

**Figure 3:**
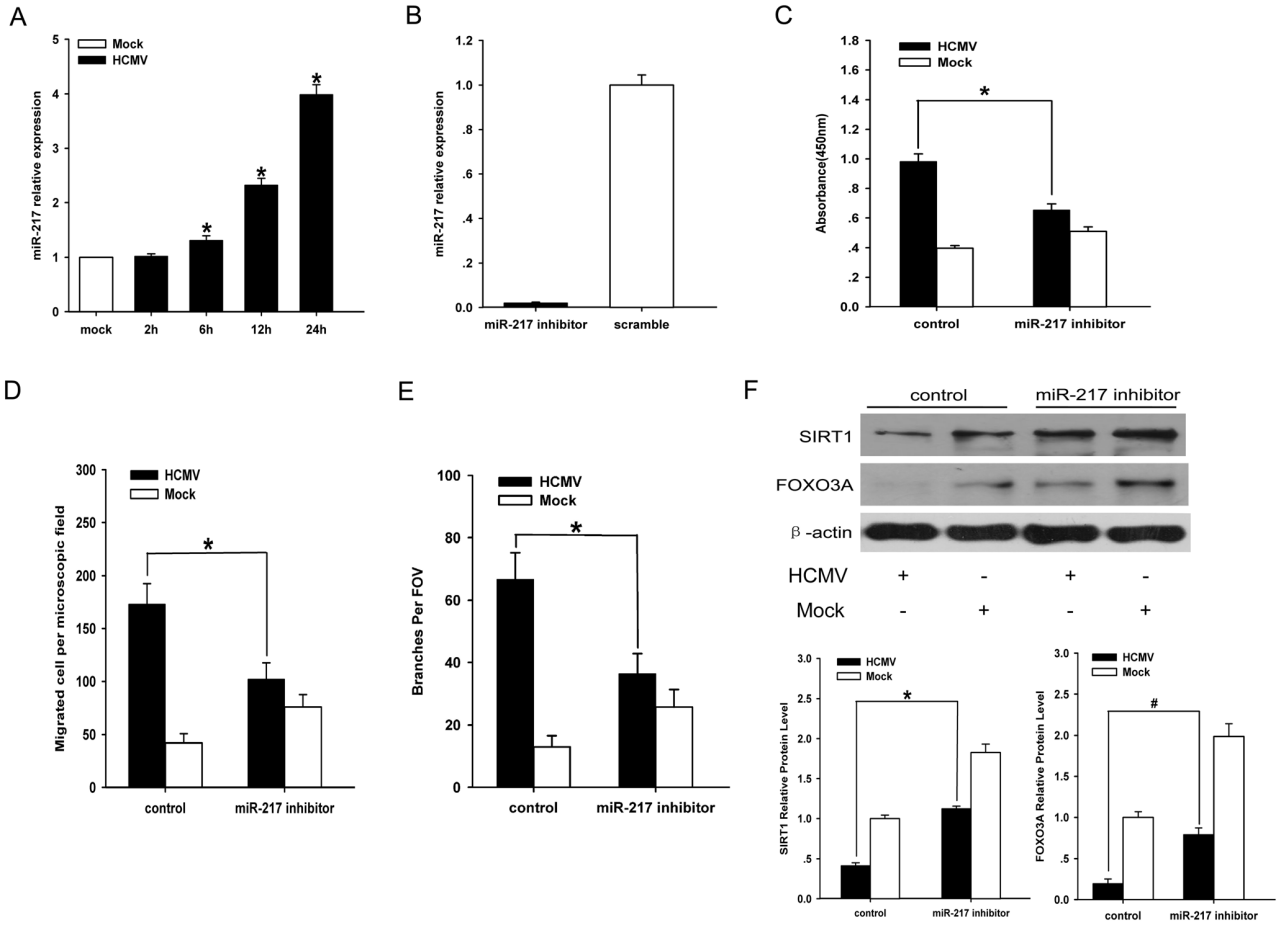
Effect of miR-217 on angiogenic activity of infected endothelial cells. A.QRT-PCR analysis of miR-217 in ECs at the indicated times after mock or HCMV infection. Mock infected ECs were harvested at 24hpi. U6 serves as an internal control. The expression of miR-217 from mock infected ECs was set as reference. B.QRT-PCR analysis of miR-217 expression in transiently transfected HUVECs. HUVECs were transiently transfected with miR-217 inhibitor or miR scramble control for 48 hours. C. Proliferation rate analysis in infected ECs in the presence or absence of anti-miR-217 at 72hpi. D. Migration analysis of ECs that were treated with the same methods as those used in Fig. 3C. Cells were fixed and stained with DAPI and quantified at 24hpi. Scale bar = 100um. E. Matrigel tube formation analysis of ECs that were treated with the same methods as those used in Fig. 3C. Cumulative sprout number of capillary-like structures was measured after 24hpi. Scale bar = 300um. F. Western blot analysis of SIRT1 and FOXO3A protein expression after transfection of miR-217 inhibitor or its negative control. ß-actin serves as loading control. SIRT1 and FOXO3A protein level from mock scramble control ECs serves as reference. Data are the mean of three independent experiments, presented as mean±SD. *P and **^#^**P<0.05.

As miR-217 is known to suppress SIRT1 protein expression by binding to SIRT1 3`UTR [16], it followed a hypothesis that HCMV, via miR-217, induces angiogenesis of ECs through inhibition of SIRT1 expression. To test this hypothesis, qRT-PCR analysis of miR-217 expression showed that the expression of miR-217 continued to accumulate in 24 hpi(Fig. 3A). Second, miR-217 inhibitor(100nM) was transfected into HUVECs before infection. Transfection efficiency of 100nM microRNA negative control can be seen Fig. S3A. Real-time PCR results showed the expression of miR-217 in HUVECs transfected with 100 nM miR-217 inhibitor compared with its scrambled control(Fig. 3B). As shown in Fig. 3C, 3D, 3E, Fig. S3B and Fig. S3C, knockdown of miR-217 reduced the proliferation rate, motility and tube formation ability of HCMV-infected ECs, but these angiogenic responses of mock-infected ECs were significantly enhanced. We next found that delivery of miR-217 inhibitor to infected ECs could alleviate SIRT1 protein expression(Fig. 3F). Since our experimental data showed that SIRT1 activated by resveratrol repressed migration and tube formation of HCMV-infected ECs, it may suggest that silence of miR-217 moderates motility and tube formation of HCMV-infected ECs through upregulation of SIRT1.

To determinate whether anti-angiogenesis of miR-217 knockdown was associated with FOXO3A expression, Western blot analysis indicated that transfection of miR-217 inhibitor enhanced expression of FOX3A protein level in infected ECs(Fig. 3F). As our experimental data showed that delivery of FOXO3A overexpression lentivirus to infected ECs reduced proliferation, migration and tube formation of infected ECs, we suggest that inhibition of miR-217 attenuated angiogenesis of infected ECs through upregulation of FOXO3A.

### 4. 4. miR-217 directly binds to FOXO3A 3`UTR

**Figure 4:**
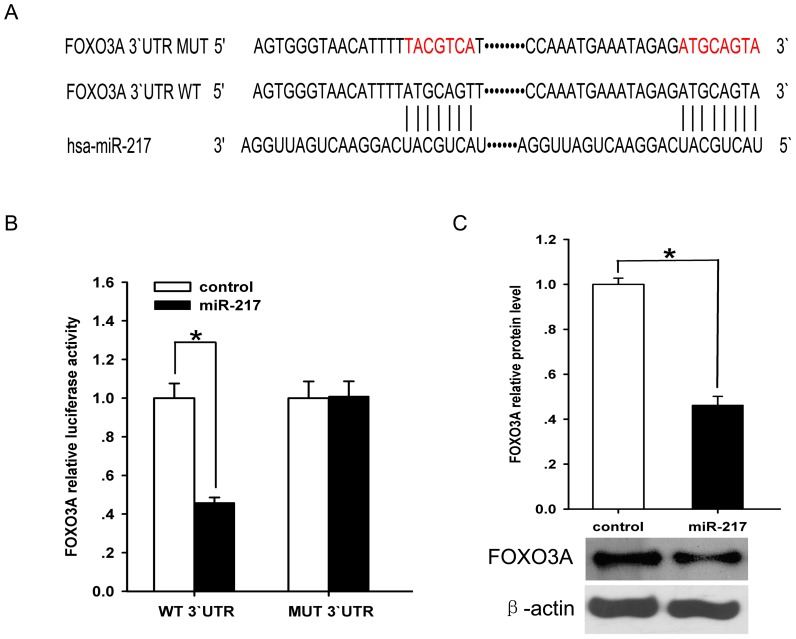
Effect of miR-217 on FOXO3A expression. A. The 3`-UTR of FOXO3A is putative target of miR-217. The seed of miR-217 displays complementarity to FOXO3A 3` UTR. Mutated nucleotides are shown in red. B. Target luciferase reporter assay using plasmids containing FOXO3A 3`UTR. The firefly luciferase activity was normalized to the Renilla luciferase activity for each sample. C. Western blot analysis of FOXO3A protein expression in miR-217 mimic delivered endothelial cells. ß-actin serves as loading control. FOXO3A protein level from miR-217 scramble control ECs serves as reference. Data represents the mean value of three independent experiments, Mean±SD. *P <0.05.

We next performed an *in silico* complementarity search using targetscan to demonstrate whether miR-217 directly binds to FOXO3A 3`UTR. such exercise revealed that 3`-untranslated regions(3`-UTR) of FOXO3A contain two binding sites for miR-217(Fig. 4A). To validate whether FOXO3A 3`-UTR is a target for miR-217, FOXO3A 3`UTR reporter luciferase assay was performed using human embryonic kidney 293 cells. Delivery of miR-217 mimic significantly altered FOXO3A 3`UTR reporter luciferase activity(Fig. 4B). Western blot analyses indicated that miR-217 negatively regulates the expression of FOXO3A protein (Fig. 4C). The data suggest that miR-217 negatively regulates the expression of FOXO3A protein by acting on FOXO3A mRNA 3`UTR.

## Discussion

In the present study, we describe a novel mechanism whereby HCMV infection, via miR-217, regulates angiogenic response of endothelial cells by altering SIRT1 and FOXO3A. We demonstrated that HCMV infection promotes angiogenesis by reduction of SIRT1 and FOXO3A. In addition, downregulation of miR-217 can attenuate the angiogenic response induced by HCMV infection. Furthermore, miR-217 was found to negatively regulate expression of FOXO3A.

Angiogenesis plays an essential role during the whole lifespan, and an imbalance of angiogenesis leads to many disorders [17]. Accumulating evidence suggests that HCMV infection is involved in angiogenesis and promotes angiogenesis by both direct and indirect mechanisms [18], and the involvement of cellular gene in angiogenic responses induced by HCMV infection has attracted much attention. For example, Fiorentini S et al. found that the induction of IL-6 and granulocyte-macrophage colony-stimulating factor(GM-CSF), as a result of HCMV infection, enhances angiogenic properties of both lymphatic ECs and blood Ecs [19]. Sara Botto et al. showed that IL-6 secreted by HCMV-infected ECs promotes angiogenesis of ECs through downstream activation of STAT-3 and survivin and reduction in caspase-3 and -7 activation [7]. SIRT1, as a member of mammalian NAD^+^-dependent deacetylase family, is an important modulator in cardiovascular functions in health and disease [14]. Potente M et al. have demonstrated that knockdown of SIRT1 is uniquely associated with loss of sprouting angiogenesis in vitro [20]. Moreover, SIRT1 mutant mice, which has genetic deletion of SIRT1 activity in the endothelium postnatally, has impaired formation of new vessels in response to ischemic stress [20]. However, the role of SIRT1 in angiogenesis induced by HCMV infection has not been extensively studied. In this experiment, we first demonstrated that SIRT1 depressed cellular motility and tube formation of HCMV-infected ECs, but promoted the angiogenic responses of mock-infected ECs. This observation may be explained as following: mock-infected inocula from MRC-5 cells lacked HCMV particle and factors secreted from virus-infected cells, therefore, ECs incubated with mock-infected inocula were under normal condition. In normal ECs, SIRT1 has been demonstrated to promote migration and tube formation [20]. By contrast, ECs incubated with HCMV-infected inocula were treated with inflammatory cytokine and virus particle. Several of these inflammatory factors, such as IL-6, IL-8, ICAM-1, have been demonstrated to induce EC motility and tube formation [7], [21]. Additionally, PDGF and TGF-ß, secreted from HCMV-infected cells, could induce angiogenesis of infected ECs [18]. These inflammatory markers, such as ICAM-1, TGF-ß and IL-6, not only have pro-angiogenic function, but also suppress SIRT1 activity induced by resveratrol [22]. On the other hand, SIRT1 has been demonstrated to have anti-inflammatory activity and depress the production of these inflammatory factors [23]. Therefore, we suggest that the loss of SIRT1 anti-inflammatory activity in HCMV-infected ECs resulted in pro-angiogenic responses. As for virus particle, HCMV has been demonstrated to prevent eNOS activation by inhibiting AKT pathway in EC [24], and Hidetaka Ota et al. have indicated that NO production by eNOS activation enhances expression of SIRT1 [25]. Thus HCMV particle may depress expression of SIRT1 in ECs. In addition, Jia Y et al. have shown that downregulation of SIRT1 enhances ICAM-1 expression in HUVEC [26], and the expression of ICAM-1 induced by HCMV infection could enhance angiogenesis of endothelial cel [27]. These experimental results suggest that downregulation of SIRT1 by HCMV infection enhanced ICAM-1 expression, thereby inducing EC motility and tube formation. This was supported by our observation. Together, our experimental data suggest that HCMV infection promoted expression of these pro-angiogenic cytokines through downregulation of SIRT1, eventually inducing angiogenesis of ECs. The precise mechanism of the action of SIRT1 still remains to be elucidated.

FOXO3a transcription factor is critical regulators of stress responses, longevity, apoptosis and oncogenesis [28]. FOXO3A suppresses vascular smooth muscle cell proliferation and neointimal hyperplasia by silence of angiogenic immediate early gene CYR61 [29]. Silence of FOXO3A gene expression can lead to upregulation of eNOS, Ang-2 and ELK-3, with a profound increase in the migratory and sprout-forming capacity of endothelial cells [15]. In this study, we found that FOXO3A impeded proliferation rate, motility and tube formation of HCMV- or mock-infected ECs. This observation is supported by other observation that silencing of endogenous FOXO3A gene expression significantly increases endothelial migration and tube formation in the Matrigel assay [14], [15].

The role of viral miRNA and host miRNA in HCMV pathogenesis also has attracted much attention. For example, HCMV encoded miR-UL112-1 is responsible for suppression of HCMV replication by multiple mechanisms, like through BclAF1 and IE72 protein associated pathways [30], [31]. HCMV-miR-US25-2 also represses HCMV replication through inhibition of eIF4A1 [32]. In contrast, HCMV-miR-UL36 induces enhancement of HCMV DNA synthesis at 24hpi by downregulation of HCMV UL138 protein [33]. In addition, our previous study showed that miR-199a-5p can enhance pro-angiogenic property of HCMV infected ECs by SIRT1/eNOS axis [34]. On the other hand, miR-217 has been demonstrated to directly bind to SIRT1 3`UTR and thus impair angiogenesis via inhibition of SIRT1 and modulation of FoxO1 (forkhead box O1) and endothelial nitric oxide synthase acetylation [16]. We therefore hypothesized that miR-217 is involved in angiogenesis induced by HCMV infection. In the present study, we showed that miR-217 is upregulated in HCMV-infected ECs, and provided a novel mechanism whereby miR-217 can benefit proliferation, migration and tube formation of HCMV-infected ECs by inhibition of both SIRT1 and FOXO3a. We further revealed that miR-217 directly targets FOXO3a mRNA 3`UTR. Together, our results strongly suggest that pro-angiogenic effect of miR-217 on infected ECs was associated with downregulation of SIRT1 and FOXO3A.

In conclusion, we have identified an endogenous inhibitor of SIRT1 and FOXO3A, miR-217, that promotes angiogenesis of HCMV-infected ECs. This work sheds new light on the regulation of angiogenesis by HCMV infection and a therapeutic strategy for preventing the pathological angiogenesis.

## Supporting Information

Figure S1 **Infection efficiency of HCMV to ECs.** Co-staining of IE-72(green) and cell nucleus(blue) was performed using immunofluorescence assay. green, FITC; blue, DAPI staining. Scale bar = 50 um.
(TIF)

Figure S2 **Effect of SIRT1 and FOXO3A on migration and tube formation of HCMV-infected ECs.** A. SIRT1 knockdown using transfection of siRNA. Immunoblot for SIRT1 and β-actin 24, 48, 72 hours after SIRT1 siRNA or its scramble control transfection in ECs showing efficient knockdown of endogenic SIRT1 protein. B. Downregulation of SIRT1 promotes migration of HCMV-infected ECs. Cell migration analysis of ECs that were treated with the same methods as those used in Fig. 2A. Cells were fixed and stained with DAPI and quantified after 24hpi. Scale bar = 100 um. C. Downregulation of SIRT1 promotes tube formation of HCMV-infected ECs. Cell tube formation analysis of ECs that were treated with the same methods as those used in Fig. 2A. Cumulative sprout number of capillary-like structures was measured after 24hpi. Scale bar = 300 um. D. FOXO3A knockdown using transfection of siRNA. Western blot analysis of FOXO3A and actin 48 and 72 hours after control or FOXO3A siRNA transfection in ECs showing efficient knockdown of endogenic FOXO3A protein. E. Overexpression of FOXO3A using lentivirus vector. Western blot analysis of FOXO3A protein expression after lentiviral overexpression of FOXO3A at MOI of 0, 10, 20. β-actin serves as loading control. F. Downregulation of FOXO3A promotes migration of HCMV-infected ECs. Cell migration analysis of ECs that were treated with the same methods as those used in Fig. 2D. Cells were fixed and stained with DAPI and quantified after 24hpi. Scale bar = 100 um. G. Downregulation of FOXO3A promotes tube formation of HCMV-infected ECs. Cell tube formation analysis of endothelial cells that were treated with the same methods as those used in Fig. 2D. Cumulative sprout number of capillary-like structures was measured after 24hpi. Scale bar = 300 um.
(TIF)

Figure S3 **Effect of miR-217 on migration and tube formation of HCMV-infected ECs.** A. Transfection efficiency of 100 nm microRNA after 6 hours. Co-staining of scrambled microRNA(red) and nucleus(blue) was performed. red, cy3; blue, DAPI staining. Scale bar = 250 nm. B. Inhibition of miR-217 depresses migration of HCMV-infected ECs. Migration analysis of ECs that were treated with the same methods as those used in Fig. 3C. Cells were fixed and stained with DAPI and quantified at 24hpi. Scale bar = 100 um. C. Inhibition of miR-217 depresses tube formation of HCMV-infected ECs. Matrigel tube formation analysis of ECs that were treated with the same methods as those used in Fig. 3C. Cumulative sprout number of capillary-like structures was measured after 24hpi. Scale bar = 300 um.
(TIF)
