# Supplementary figures and images for: MicroRNA-217 Promotes Angiogenesis of Human Cytomegalovirus-Infected Endothelial Cells through Downregulation of SIRT1 and FOXO3A
Source: PLoS One. 2013 Dec 20;8(12):e83620. doi: 10.1371/journal.pone.0083620 (PMC3869804; doi:10.1371/journal.pone.0083620)

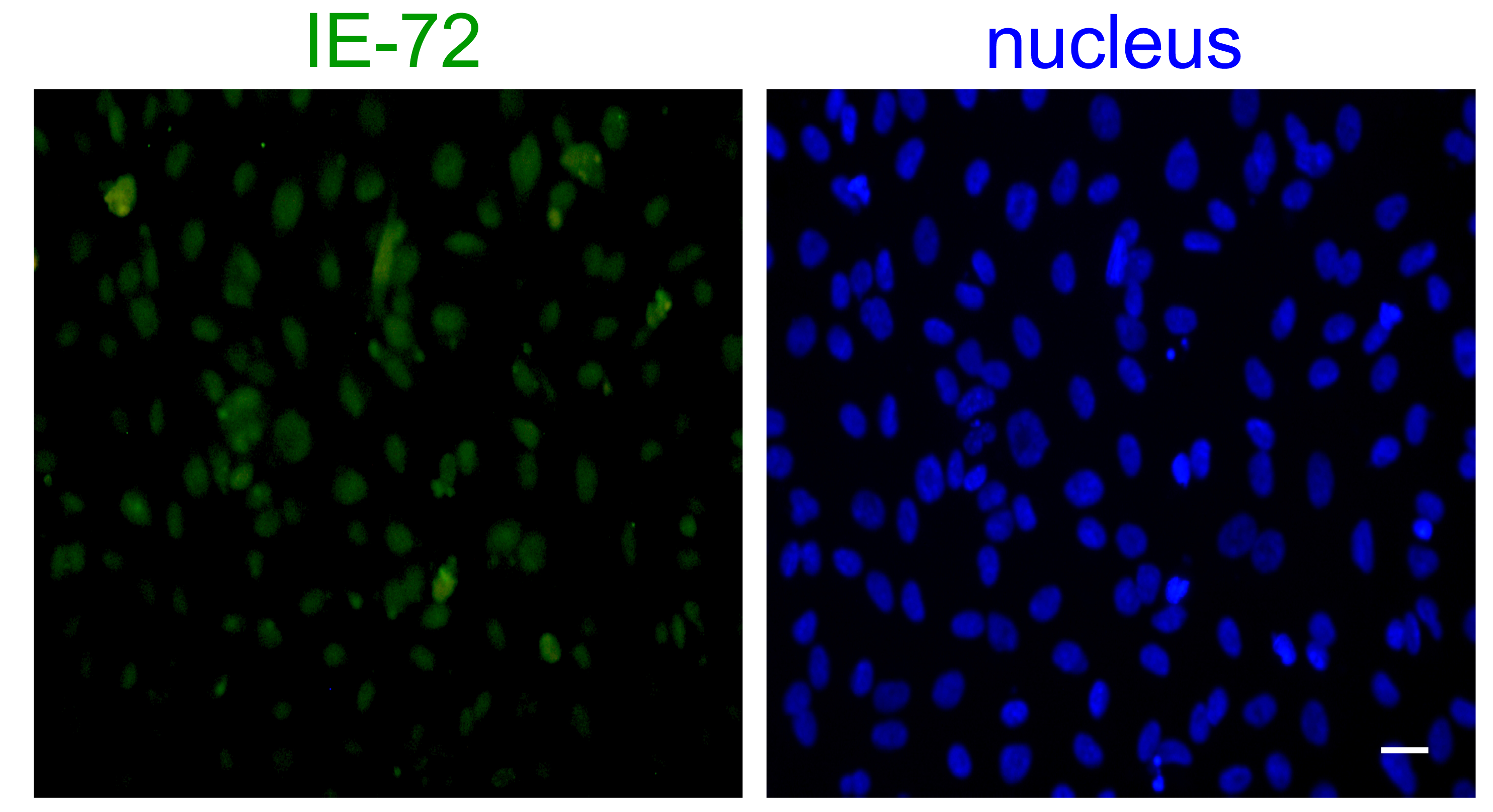

Supplement: Figure S1 — Infection efficiency of HCMV to ECs. Co-staining of IE-72(green) and cell nucleus(blue) was performed using immunofluorescence assay. green, FITC; blue, DAPI staining. Scale bar = 50 um. (TIF) [file pone.0083620.s001.tif]

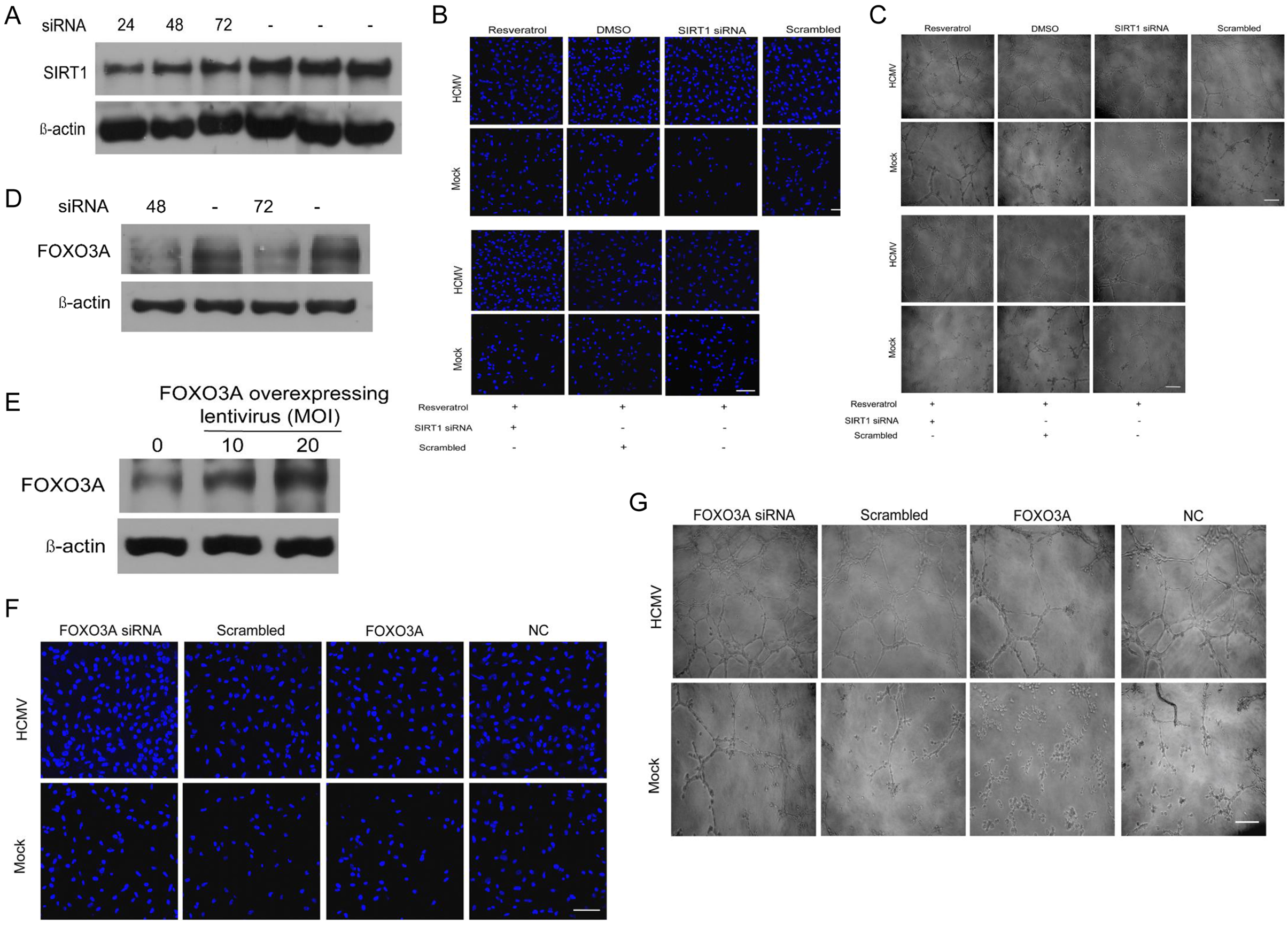

Supplement: Figure S2 — Effect of SIRT1 and FOXO3A on migration and tube formation of HCMV-infected ECs. A. SIRT1 knockdown using transfection of siRNA. Immunoblot for SIRT1 and β-actin 24, 48, 72 hours after SIRT1 siRNA or its scramble control transfection in ECs showing efficient knockdown of endogenic SIRT1 protein. B. Downregulation of SIRT1 promotes migration of HCMV-infected ECs. Cell migration analysis of ECs that were treated with the same methods as those used in Fig. 2A. Cells were fixed and stained with DAPI and quantified after 24hpi. Scale bar = 100 um. C. Downregulation of SIRT1 promotes tube formation of HCMV-infected ECs. Cell tube formation analysis of ECs that were treated with the same methods as those used in Fig. 2A. Cumulative sprout number of capillary-like structures was measured after 24hpi. Scale bar = 300 um. D. FOXO3A knockdown using transfection of siRNA. Western blot analysis of FOXO3A and actin 48 and 72 hours after control or FOXO3A siRNA transfection in ECs showing efficient knockdown of endogenic FOXO3A protein. E. Overexpression of FOXO3A using lentivirus vector. Western blot analysis of FOXO3A protein expression after lentiviral overexpression of FOXO3A at MOI of 0, 10, 20. β-actin serves as loading control. F. Downregulation of FOXO3A promotes migration of HCMV-infected ECs. Cell migration analysis of ECs that were treated with the same methods as those used in Fig. 2D. Cells were fixed and stained with DAPI and quantified after 24hpi. Scale bar = 100 um. G. Downregulation of FOXO3A promotes tube formation of HCMV-infected ECs. Cell tube formation analysis of endothelial cells that were treated with the same methods as those used in Fig. 2D. Cumulative sprout number of capillary-like structures was measured after 24hpi. Scale bar = 300 um. (TIF) [file pone.0083620.s002.tif]

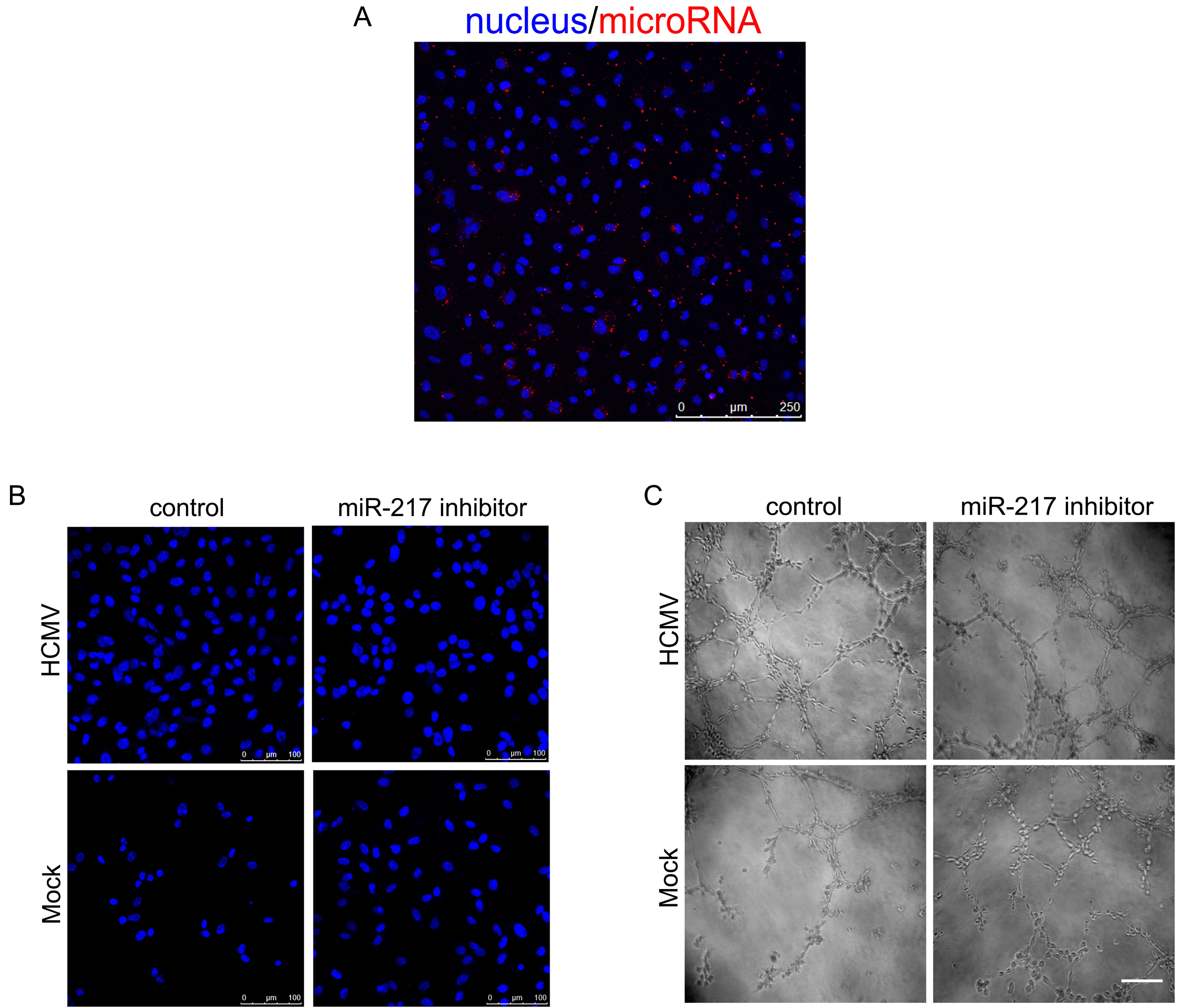

Supplement: Figure S3 — Effect of miR-217 on migration and tube formation of HCMV-infected ECs. A. Transfection efficiency of 100 nm microRNA after 6 hours. Co-staining of scrambled microRNA(red) and nucleus(blue) was performed. red, cy3; blue, DAPI staining. Scale bar = 250 nm. B. Inhibition of miR-217 depresses migration of HCMV-infected ECs. Migration analysis of ECs that were treated with the same methods as those used in Fig. 3C. Cells were fixed and stained with DAPI and quantified at 24hpi. Scale bar = 100 um. C. Inhibition of miR-217 depresses tube formation of HCMV-infected ECs. Matrigel tube formation analysis of ECs that were treated with the same methods as those used in Fig. 3C. Cumulative sprout number of capillary-like structures was measured after 24hpi. Scale bar = 300 um. (TIF) [file pone.0083620.s003.tif]
